# Correction: The economic burden of multimorbidity: Protocol for a systematic review

**DOI:** 10.1371/journal.pone.0316571

**Published:** 2024-12-26

**Authors:** Amrit Banstola, Nana Anokye, Subhash Pokhrel

There are errors in the Funding statement. The correct Funding statement is as follows: This study is part of the first author’s PhD studies funded by the Department of Health Sciences at Brunel University of London. This research is partly funded by the NIHR (NIHR203257) using UK international development funding from the UK Government to support global health research. The views expressed in this publication are those of the author(s) and not necessarily those of the NIHR or the UK government.
