# Supplementary material for: The economic burden of multimorbidity: Protocol for a systematic review
Source: PLoS One. 2024 May 2;19(5):e0301485. doi: 10.1371/journal.pone.0301485 (PMC11065216; doi:10.1371/journal.pone.0301485)
Supplement: S1 Appendix — (DOCX) [file pone.0301485.s002.docx]

**Supporting Information file S1:**

**A draft SCOPUS search strategy**

(TITLE(cost* OR financ* OR economic* OR expenditure* OR expense* OR spend* OR pay*)) AND (((TITLE-ABS (comorbid* OR co-morbid* OR multimorbid* OR multi-morbid* OR "chronic disease")) OR (TITLE-ABS (multidisease* OR multi-disease* OR multi-condition* OR multicondition*)) OR (TITLE-ABS((multi OR multiple) W/2 (morbid* OR ill* OR disease* OR condition* OR syndrom* OR diagnos* OR disorder*))) OR (TITLE-ABS((cooccur* OR co-occur* OR coexist* OR co-exist* OR multipl* OR concord* OR discord*) W/3 (disease* OR ill* OR care OR condition* OR disorder* OR health* OR medication* OR symptom* OR syndrom* OR morbid*))) OR (TITLE-ABS((polypatholog* OR poly-patholog* OR polymorbid* OR poly-morbid* OR multipatholog* OR multi-patholog* OR pluripatholog* OR pluri-patholog* OR concurrent) W/2 (disease* OR illness* OR condition* OR diagnosis OR morbid*))) OR (TITLE-ABS(chronic* W/1 (disease* OR ill* OR care OR condition* OR disorder* OR health* OR medication* OR syndrom* OR symptom*))) OR (TITLE-ABS(polypharmac* OR poly-pharmac* OR polymedicat* OR poly-medicat*)))) SUBJAREA(MEDI OR NURS OR DENT OR HEAL OR MULT) AND ( LIMIT-TO ( SRCTYPE,"j" ) ) AND ( LIMIT-TO ( DOCTYPE,"ar" ) ) AND ( LIMIT-TO ( LANGUAGE,"English" ) )
