# Supplementary material for: The economic burden of multimorbidity: Protocol for a systematic review
Source: PLoS One. 2024 May 2;19(5):e0301485. doi: 10.1371/journal.pone.0301485 (PMC11065216; doi:10.1371/journal.pone.0301485)
Supplement: Attachment — Submitted filename: Response to Reviewers.docx [file pone.0301485.s003.docx]

**Response to Reviewers**

Dear Editor,

Re: Manuscript ID (PONE-D-23-09002)

Title: The economic burden of multimorbidity: protocol for a systematic review

We would like to express our gratitude for the time and effort that you and the reviewers have invested in our manuscript titled "The economic burden of multimorbidity: protocol for a systematic review." Your valuable feedback has provided us with insights to enhance the quality of our work, and we appreciate the opportunity to respond to the points raised.

Below we have addressed each of the points raised by the reviewers in a comprehensive manner:

**REVIEWER 1**

**Comment 1: The authors have reviewed the available literature and the findings have been presented well. They have clearly presented the findings and limitations of each literature which is well appreciated. It would be better if the authors could state the rationale for performing this systematic review. If would be better if the readers could clearly understand what this systematic review will going to add in the literature that are already available.**

Response: Thank you for the comment. We acknowledge the importance of explicitly stating the rationale for conducting our systematic review and elucidating the unique contribution it makes to the existing literature. To address this concern, we have now improved the introduction section of our manuscript on page 5 by providing a rationale behind undertaking this systematic review. By highlighting the specific aspects that our systematic review will address, such as synthesizing diverse research, capturing various disease combinations, considering different cost components, and providing an in-depth understanding of the intricate relationship between multimorbidity and healthcare costs, we aim to clearly convey the unique value that our study adds to the existing body of literature.

**Comment 2: The authors need to declare the source of financial and non-financial support for this systematic review. Although the authors have mentioned that they received non-financial support from the university library in finalizing the search strategy, the authors need to declare whether or not they have received any financial support for conducting this review.**

Response: Thank you for your comment. We have clearly stated the funding information in the financial disclosure section of the submission system as required by the journal. The [submission guidelines](https://journals.plos.org/plosone/s/submission-guidelines#loc-acknowledgments) suggested the following:

*“Do not include funding sources in the Acknowledgments or anywhere else in the manuscript file. Funding information should only be entered in the financial disclosure section of the submission system.”*

For your information, we have stated the following financial disclosure in the submission system:

*“The author(s) received no specific funding for this work. This study is part of the first author's PhD project and that the first author is sponsored by the Department of Health Sciences at Brunel University London.”*

**Comment 3. I would suggest the authors to elaborate more on the discussion section.**

Response: Thank you for your suggestion to elaborate more on the discussion section. We have now added few sentences in the first paragraph of the Discussion section on page 14.

**REVIEWER 2**

**Comment 1: The protocol states that searches will be done until March 2023- the timeline has already been passed- has the searches been already completed or still to be done. It needs to adjust accordingly.**

Response: Thank you for highlighting this. We acknowledge that the stated timeline has now been passed. But we have not yet carried out the searches. We have therefore, amended this to August 2023 in the revised manuscript.

**Comment 2: The economic burden of multimorbidity very much depends on what kind of health financing system a particular country has. Although the paper states that it will review across the countries, but the one of the important factors that affects the economic burden is the health systems architect of that particular country. So, the systematic review should consider income level and health financing system of the country in concern for generalization of the findings from countries with varying income level and health financing system.**

Response: We appreciate your insightful comment to consider income level and health financing system of the country in the analysis. We agree that it is an important consideration when interpreting and generalizing our findings. In response to your valuable feedback, we added the following sentence in the ‘Data analysis and synthesis’ section on page 12.

“The findings will be presented as the percentage of the gross national income per capita alongside the health financing systems of the country.”

We have also added the following texts to highlight the importance of such analysis in the ‘Discussion’ section on page 14.

“By analysing the gross national income per capital and the health financing systems of each country, this review will provide a deeper understanding of how these factors influence the economic burden of multimorbidity across diverse nations. This understanding also holds significance in facilitating the interpretation and generalization of the findings to various healthcare contexts.”

**Comment 3: Although various indices have been included as the inclusion criteria for the review, it would be good to elaborate on what kind of weighted indices will be used as the methodology for economic burden analysis as stated in the objectives. Methodology and reporting quality could have major implication on the reported costs. The protocol is less explicit on how the ‘methodological and reporting quality’ [second objective] will interact on the analysis of the economic burden [first objective]. This is important to clarify in the protocol.**

Response: Thank you for your valuable comment concerning the use of weighted indices. To clarify, we plan to present the economic burden for each available weighted index, rather than adhering to a specific index as the sole methodology for economic burden analysis. This approach allows us to consider a variety of perspectives and capture the multidimensionality of economic burden assessments.

We appreciate that methodology and reporting quality could have major implication on the reported costs and that is what we are going to showcase through this review. In the ‘Risk of bias assessment’ section, we have outlined our strategy to evaluate the methodological quality of included studies using a well-established appraisal checklist appropriate to the study design. Additionally, we have emphasised that this quality assessment will aid the interpretation of the analysis, but it will not determine exclusion. We have amended the first sentence of ‘Data analysis and synthesis’ section to make it clear. The revised sentences now explicitly state our intention to provide a narrative synthesis due to the expected heterogeneity and insufficient detail in the included studies.

**Comment 4: Economic burden could be to the patients versus to the overall health systems in itself which needs to be clarified in terms of scope of the study. If it is to focus on economic burden on individual patient, it has strong association with how the health financing system is designed which needs to be clarified.**

Response: Thank you for your valuable feedback. We completely agree that the economic burden can encompass both patients and overall health systems. To address this aspect, we have taken steps to clarify the scope of our study.

In the 'Data analysis and synthesis' section on page 12, we have explicitly stated that we will organize our findings based on the ‘perspectives adopted’, which could range from the patient perspective to the health system perspective. Furthermore, we have emphasized that we will report the ‘mean annual costs per patient’, indicating the economic burden on individual patients, particularly those related to direct medical costs and direct non-medical costs. Additionally, we will present any available information on indirect costs, shedding light on burdens beyond individual patients. In this manner, our study intends to capture the multifaceted nature of the economic burden.

We also appreciate your insight about the association between economic burden on individual patients and the design of the health financing system. We have taken your suggestion into account and made appropriate amendments to the manuscript (also as part of your comment #2).

**Comment 5: In the background, it states that ‘’there might have been several studies 77 within this time that explored the economic impact of multimorbidity’’ indicating to review those papers. However, the paper has stated that it will exclude papers on ‘economic evaluation’. Please present it with logical linkage for consistency and clarity.**

Response: Thank you for bringing up this point. We want to clarify that our review will specifically focus on the economic impact of multimorbidity. As you correctly noted, we will exclude papers categorised as ‘economic evaluation’ because they often involve comparing the costs and benefits of interventions and strategies aimed at preventing, managing or controlling multimorbidity. Thus, there inclusion is beyond the scope of this review. To make it clear, we have made the necessary adjustment on page 7. We added the phrase ‘which compares two or more interventions’ after ‘economic evaluation studies’ to explicitly highlight the distinction and reinforce our review’s exclusive emphasis on the economic impact of multimorbidity.

We have carefully addressed the editor's and reviewers' suggestions, revising the manuscript accordingly. Our revised manuscript is available as two separate files: 'Revised Manuscript with Track Changes,' which highlights alterations made, and 'Manuscript,' the unmarked version. The revision has enhanced the study's quality and rigor. We appreciate the review process and valuable insights from the editor and reviewers. Kindly consider our revised version. We remain open to further suggestions.

Thank you for your time. We await your decision eagerly.

Sincerely,

Amrit Banstola

On behalf of the review team
