# Supplementary material for: The economic burden of multimorbidity: Protocol for a systematic review
Source: PLoS One. 2024 May 2;19(5):e0301485. doi: 10.1371/journal.pone.0301485 (PMC11065216; doi:10.1371/journal.pone.0301485)
Supplement: Attachment — Submitted filename: Review feedback.docx [file pone.0301485.s006.docx]

**Feedback**

Thank-you for the opportunity to review the presented manuscripts entitled “*The economic burden of multimorbidity: protocol for a systematic review*”.

The study aims to investigate an important subject-it stands to add valuable health economic data/information in the field of public health and inform key policy decision making discussions aimed at addressing global/country specific disease burden response.

The authors presented technically sound methods/approach for answering the study question. Authors applied conventional standard (E.g., PRISMA -P tool) to inform items to present/address in a systematic review protocol.

**Recommendation:** Protocol to be considered for publication once the below comments (categorized as non-major) have been addressed.

**Specific comments:**

**Introduction**

*Line 44*: multiple instead of larger number

*Line 63*: It is difficult to interpret the "The single chronic disease" Please expand.

**Risk of bias assessment**

*Line 182*: The risk bias assessment is fairly articulated. Please confirm if these s happen at the outcome or study level or both.

**Data Analysis and synthesis**

*Line 210-211*: Whilst I appreciate the reporting of the findings by gross national income per capita , please note that this measure may not necessarily reflect the countries/nationals financial capacity to absorb the financial burden posed by multi-morbidity. The interpretation should be in the context of other economic indices e.g., Gini coefficient ranking. Countries with high income in-equality may still present with a high GD per capita yet a majority of the households/individuals would be living below poverty line. This can be masked when GD per capita is interpreted in-silos.

*Line 217*: Indirect costs will shed light on economic burden beyond the health care provider, specifically direct and indirect cost provide societal costs.

**Assessing the quality of evidence**

*Line 224-230*: Whilst the grading of recommendation Assessment Development and Evaluation may not be appropriate - Please describe how this study will assess the strength of body of evidence that shall be presented.

**Discussion**

*Line 244*: Can the authors specify how they intend to analyse the countries' health financing systems. Does this mean there will be a separate analysis/assessment of the respective HF systems functioning /performance. This part is not clear. Please expand for clarity

**Implications for research, practice or policy**

*Line 295*: The study is not assessing costs of inaction. However, understanding the magnitude of multi-morbidity can be an advocacy tools for strengthening disease prevention programs.

**Dissemination plans**

Line 279: Who will be your targeted audience for the webinar?

**General:**

The authors did not articulate where/how data underlying the findings will be made available
