# Supplementary material for: The economic burden of multimorbidity: Protocol for a systematic review
Source: PLoS One. 2024 May 2;19(5):e0301485. doi: 10.1371/journal.pone.0301485 (PMC11065216; doi:10.1371/journal.pone.0301485)
Supplement: Attachment — Submitted filename: Response to Reviewers.docx [file pone.0301485.s007.docx]

**Response to Reviewers (2^nd^ Revision)**

Dear Editor,

Re: Manuscript ID (PONE-D-23-09002)

Title: The economic burden of multimorbidity: protocol for a systematic review

We would like to express our gratitude for the time and effort that you and the reviewers have invested in our manuscript titled "The economic burden of multimorbidity: protocol for a systematic review." Your valuable feedback has provided us with insights to enhance the quality of our work, and we appreciate the opportunity to respond to the points raised.

Below we have addressed each of the points raised by the reviewers in a comprehensive manner:

**Reviewer 3**

| **Reviewer comment** | **Author’s response** |
| --- | --- |
| This is a well-designed protocol study. But, since comorbidity includes a wide range of diseases, it seems that not limiting the study to a range of diseases (based on the global burden of disease or...), challenges the validity of the study. | Thank you for your valuable feedback on our protocol. We acknowledge the wide range of disease encompassed by comorbidity and understand the importance of ensuring the validity of our study. In response to your concern, we plan to align our study with the [UK NIHR Global Health Research Centre for Multiple Long-Term Conditions](https://www.fundingawards.nihr.ac.uk/award/NIHR203257) at the time of write-up. By doing so, we aim to provide a more targeted and comprehensive analysis while maintaining the validity and relevance of our findings. |
| However, some recommendations that may be helpful in improving the study design were included in the text of the article as comments. | Thank you very much for providing constructive comments. We have addressed each of your comments below in the revised version. |
| page 6: Types of costs in the health sector include direct medical, direct non medical and indirect cost. Therefore, the cost as an inclusion criterion includes the three types of costs mentioned | Thank you for your comments on that. We have incorporated the indirect costs and included them within the costs as an inclusion criterion. The revised manuscript includes the three types of costs as you have suggested, on page 7. |
| page 7- Some full economic evaluation studies report all cost in detail, Why are they among the exclusion criteria? | We thank the reviewer for this comment. We acknowledge that some full economic evaluation studies report all cost in detail, and these studies could provide valuable information on the economic burden of multimorbidity. However, we decided to exclude these studies from our systematic review for the following reasons: Full economic evaluation studies typically compare the costs and outcomes of two or more interventions, rather than estimating the total costs of a disease or condition. Therefore, the costs reported in these studies may not reflect the full economic burden of multimorbidity, but rather the incremental costs of a specific intervention or treatment strategy. Full economic evaluation studies may not provide sufficient details on the sources, methods, and components of costs, especially when the focus is on the outcomes or the cost-effectiveness ratios. This may limit the transparency, reproducibility, and generalizability of the cost estimates, and make it difficult to synthesize and analyse the economic burden across different studies. Based on these considerations, we chose to include only observational studies (cross-sectional, cohort, case-control) and costing/cost-of-illness studies or economic burden studies including model-based studies in our protocol. These studies are more likely to provide comprehensive and comparable estimates of the economic burden of multimorbidity, which is the main objective of our systematic review. We agree that excluding full economic evaluation studies is a limitation of our review, therefore we have stated it in the ‘Strengths and limitations of the study design’ section, on pages 15-16, of the revised manuscript.  As part of the first author's PhD, a separate systematic review based on full economic evaluation of interventions for the management of people with multimorbidity is also being conducted. |
| page 11- subgroup analysis did not mentioned. it help to better understanding and comparing different condition? | Thank you for your suggestion regarding subgroup analysis. We have included a section on subgroup analysis in the revised version of the manuscript on pages 12-13, under the 'Data analysis and synthesis' heading. This section outlines our approach to subgroup analysis, including the specific subgroups considered. |

**Reviewer 4**

| **Reviewer comment** | **Author’s response** |
| --- | --- |
| I read your protocol thoroughly. This is an interesting systematic review protocol. It is written quite well and there are not many comments on it from my side at the moment. I would like to provide a couple of supportive comments to make the protocol more robust. | Thank you for your positive feedback on our systematic review protocol. We greatly appreciate your supportive comments. |
| In Introduction section, line 102, you have included AMS in brackets, not sure why. Probably AMS shouldn’t be in brackets, please check it. | Thank you for bringing this to our attention. We have revised the manuscript accordingly to ensure AMS is appropriately integrated into the text without the use of brackets. |
| Please revise the inclusion of studies published date within November 2023 as it is already middle of the November (lines 147/8), as August has already gone. | Thank you for bringing this to our attention. We have revised the timeline for inclusion of studies to March 2024, as indicated in both the Abstract and on page 10 of the revised manuscript. This adjustment ensures that our review encompasses the most up-to-date research available. |
| In the section ‘Assessing the Quality of Evidence’ (lines 224-230), you have talked only about GRADE, which you said not suitable for this review. If there any other suitable tool, you can talk about that to assess the quality of evidence. If not remind the readers about your RoB tools for ‘quality assessment’ of papers and write down how these tools will be helpful to make sure your synthesised evidence is robust. | Thank you for raising this important point. After careful consideration, we have found that there are no other suitable tools to assess the quality of evidence for our review. As suggested, we have reminded readers about our Risk of Bias (RoB) assessment tools for quality assessment of papers. In the revised manuscript, on page 13, we have elaborated on how these tools will be instrumental in ensuring the robustness of our synthesised evidence. |
| Other contents look good. Best wishes for your review. I am looking forward to reading your final systematic review report. | Thank you for your positive feedback and well wishes! We appreciate your support and look forward to delivering the final systematic review report. |

**Reviewer 5**

| **Reviewer comment** | **Author’s response** |
| --- | --- |
| Thank-you for the opportunity to review the presented manuscripts entitled “The economic burden of multimorbidity: protocol for a systematic review”.  The study aims to investigate an important subject-it stands to add valuable health economic data/information in the field of public health and inform key policy decision making discussions aimed at addressing global/country specific disease burden response.  The authors presented technically sound methods/approach for answering the study question. Authors applied conventional standard (E.g., PRISMA -P tool) to inform items to present/address in a systematic review protocol.  Recommendation: Protocol to be considered for publication once the below comments (categorized as non-major) have been addressed. | We appreciate your positive feedback on the importance of our study's subject matter and the potential impact of our research on health economics and public health policy. We have carefully considered and addressed each of your comments in the revised manuscript. |
| Introduction, Line 44: multiple instead of larger number | Thank you for your suggestion. We have used 'multiple' instead of 'larger number' on page 3, line 44 of the revised manuscript. |
| Introduction, Line 63: It is difficult to interpret the "The single chronic disease" Please expand. | Thank you for your suggestion. We have now amended the sentence in the revised manuscript on page 4, lines 63-64 for improved clarity. The amended sentence reads as follows: 'The existing healthcare systems globally have a single-disease focus, aiming to treat specific chronic conditions.' |
| Risk of bias assessment, Line 182: The risk bias assessment is fairly articulated. Please confirm if these s happen at the outcome or study level or both. | Thank you for your comment regarding the risk of bias assessment in our manuscript. To address your question, the risk of bias assessment outlined in our manuscript applies at the study level rather than the outcome level. We have clarified this point in the revised manuscript section 'Risk of bias assessment', on page 11, to avoid any confusion. |
| Data Analysis and synthesis, Line 210-211: Whilst I appreciate the reporting of the findings by gross national income per capita, please note that this measure may not necessarily reflect the countries/nationals financial capacity to absorb the financial burden posed by multi-morbidity. The interpretation should be in the context of other economic indices e.g., Gini coefficient ranking. Countries with high income in-equality may still present with a high GD per capita yet a majority of the households/individuals would be living below poverty line. This can be masked when GD per capita is interpreted in-silos. | Thank you for your insightful comment. We appreciate your suggestion to consider other economic indices, such as the Gini coefficient, to provide a more comprehensive interpretation of our results. To address this, we have considered incorporating the Gini coefficient into our data analysis and synthesis, on page 12 of the revised manuscript. Specifically, we will explore how factors such as income inequality may influence the interpretation of our findings on gross national income per capita. By contextualising our results within a broader economic framework, we aim to provide a more nuanced understanding of the financial implications of multimorbidity for different populations. |
| Data Analysis and synthesis, Line 217: Indirect costs will shed light on economic burden beyond the health care provider, specifically direct and indirect cost provide societal costs. | We appreciate the reviewer’s insightful feedback. You are absolutely correct that indirect costs extend beyond the health care sector and have broader societal implications. In our revised manuscript, on pages 12-13, we have emphasized that both direct and indirect costs contribute to the overall economic burden associated with the disease. By highlighting these aspects, we aim to provide a more comprehensive understanding of the economic impact beyond individual patients. |
| Assessing the quality of evidence, Line 224-230: Whilst the grading of recommendation Assessment Development and Evaluation may not be appropriate - Please describe how this study will assess the strength of body of evidence that shall be presented. | Thank you for your comment. A similar comment was raised by another reviewer (Reviewer 4), who suggested a method to address it. Following Reviewer 4's suggestion, we have revised this section of our manuscript, as detailed on page 13 of the updated version. We believe that these changes enhance the clarity and robustness of our approach to evaluating the strength of evidence. |
| Preliminary timeframe, Table 2, page 13: only a week for dissemination sound like a gross underestimation. | Thank you for bringing this to our attention. We have reviewed the timeline and extended the duration for dissemination by allocating two to four weeks to ensure sufficient time for these important activities. |
| Discussion, Line 244: Can the authors specify how they intend to analyse the countries' health financing systems. Does this mean there will be a separate analysis/assessment of the respective HF systems functioning /performance. This part is not clear. Please expand for clarity | Thank you for your comment. Our analysis will not involve a separate assessment of the respective health financing systems' functioning and performance. Instead, we will utilise data from the WHO Health Financing Dashboard, which includes information on key aspects of health financing systems such as funding sources, health spending, and other health financing indicators. To improve clarity on this point, we have expanded upon our methodology in the revised manuscript, specifically on page 12. Additionally, we have revised the sentence in the 'Discussion' section, on page 14 of the manuscript for improved clarity. |
| Implications for research, practice or policy, Line 295: The study is not assessing costs of inaction. However, understanding the magnitude of multi-morbidity can be an advocacy tools for strengthening disease prevention programs. | Thank you for your comment. We have revised the sentence on page 15 of the revised manuscript. The new sentences read as follows:  "In other words, the review's findings are crucial in expanding our understanding of the economic burden of multimorbidity. These insights can serve as an advocacy tool to strengthen disease prevention programmes and guide informed decision-making on resource allocation and health service organisation through the design and implementation of cost-effective interventions." |
| Dissemination plans, Line 279: Who will be your targeted audience for the webinar? | Thank you for your question regarding the dissemination plans outlined in our manuscript. The targeted audience for the webinar will primarily include healthcare professionals, policymakers, researchers, and other stakeholders involved in multiple long-term conditions. We have included this sentence in the 'Dissemination plans' section on page 16, lines 309-314 of the revised manuscript. |
| General: The authors did not articulate where/how data underlying the findings will be made available | Thank you for your comment. We appreciate your concern regarding data availability. As part of the manuscript submission process, information on data availability is included in the PDF generated by the system as evidence. For protocols lacking pilot or preliminary data, the journal strongly encourages authors to specify how they intend to share research data from their study upon completion or publication. Therefore, we have addressed data availability in a dedicated section of our manuscript titled 'Dissemination Plans', where we outline our intentions regarding data sharing. Additionally, within the manuscript submission system, we have clearly indicated where the data can be located in full sentences. Specifically, we have used the sample text recommended by the journal, which states, "No datasets were generated or analysed during the current study. All relevant data from this study will be made available upon study completion. |

We have carefully addressed the editor's and reviewers' suggestions, revising the manuscript accordingly. Our revised manuscript is available as two separate files: 'Revised Manuscript with Track Changes,' which highlights alterations made, and 'Manuscript,' the unmarked version. The revision has enhanced the study's quality and rigor. We appreciate the review process and valuable insights from the editor and reviewers. Kindly consider our revised version. We remain open to further suggestions.

Thank you for your time. We await your decision eagerly.

Sincerely,

Amrit Banstola

On behalf of the review team
